# Effectiveness of Tranexamic Acid in Reducing Postoperative Blood Loss in Patients Undergoing Off-Pump Coronary Artery Bypass Grafting

**DOI:** 10.7759/cureus.11924

**Published:** 2020-12-05

**Authors:** Prashant Khadanga, Muralidhar Kanchi, Pallavi Gaur

**Affiliations:** 1 Anaesthesiology, Narayana Institute of Cardiac Sciences, Bangalore, IND; 2 Anaesthesiology, BYL Nair Charitable Hospital, Mumbai, IND

**Keywords:** tranexemic acid, bleeding, postoperative blood loss, off-pump coronary artery bypass grafting (op-cabg)

## Abstract

Background

Off-pump coronary artery bypass grafting (OP-CABG) is an accepted surgical option in treating ischemic heart disease and has proven safer than traditional on-pump CABG in terms of reducing perioperative bleeding, coagulopathy, avoiding cardiopulmonary bypass machine and its related morbidity. However, there is evidence that shows the risk of bleeding in OP-CABG due to surgical trauma, heart manipulations, and heparin-protamine exposure. We aim to evaluate the effectiveness of tranexamic acid (TxA) in reducing blood loss and related perioperative complications in patients undergoing OP-CABG.

Method

An individual matched cohort study was conducted at a cardiac centre over a period of one year. We enrolled a total of 60 patients undergoing OPCABG in our study. The basic strategy was to enroll every possible intervention patient until the desired sample size (30 in each group) was achieved and then to select and enroll controls, using a prospective individual matching strategy. Preoperative cardiac risk evaluation was done using the European System for Cardiac Operative Risk Evaluation II (EuroSCORE II) in both groups. The intervention group (I) received TxA 10 mg/kg over 10 minutes at the time of induction while the control group (C) did not receive any TxA. Postoperative blood loss was measured by observing chest drain output 24 hourly till the chest drain tube was removed. Perioperative complications were also recorded.

Results

Demographics and baseline characteristics were comparable among groups (p > 0.05). The mean volume of postoperative blood loss in the I group at 24 hours and 48 hours were 352.67 ml and 86.83 ml, respectively. On the other hand, in the C group, the mean volume of postoperative blood loss was 602.00 ml and 166.3 ml. The data showed a statistically significant difference in the postoperative chest drainage output between the groups (unpaired t-test, p < 0.05) and exhibiting a significant reduction in postoperative blood loss in the I group. However, there was no significant difference in blood transfusion requirements in both of the groups (Mann Whitney U test, p > 0.05). The mean duration of postoperative complications, inotropic support, intermittent positive pressure ventilation, intensive care, and hospital stay were also comparable depicting no significant effect of TxA on reducing the perioperative morbidity.

Conclusion

This study showed the significance of TxA in reducing bleeding in the postoperative period in patients undergoing OP-CABG.

## Introduction

Coronary artery disease (CAD) is one of the most common causes of morbidity and mortality across nations [1]. Off-pump coronary artery bypass grafting (OP-CABG) has gained more popularity than traditional on-pump CABG in reducing bleeding and coagulopathy by eliminating the use of cardiopulmonary bypass (CPB) machine and avoiding its related complications [2]. Although OP-CABG carries less risk of bleeding, the complications due to haemorrhage are not completely eliminated and patients might need a blood transfusion. It has been proposed that surgical traumas such as sternotomy, pericardiotomy, graft harvesting, manipulation of heart, exposure to heparin and protamine will cause activation of the fibrinolytic pathway and lead to more bleeding [2]. Moreover, blood loss and subsequent transfusions are associated with major perioperative morbidity and mortality in cardiac surgeries [2,3]. Antifibrinolytics are used in reducing perioperative bleeding and transfusion requirements in both cardiac and non-cardiac surgeries. Tranexamic acid (TxA), a synthetic antifibrinolytic and a lysine analogue has been shown to reduce blood loss in on-pump CABG. However, only a few studies on OP-CABG have reported the effectiveness of TxA in reducing postoperative bleeding with inconsistent findings on blood transfusion requirements [4]. The aim of this study was to evaluate the effects of TxA on postoperative bleeding and related complications as well as resource utilisation.

## Materials and methods

This was a prospective matched cohort study conducted over a period of one year at a tertiary care cardiac centre after approval from the Institutional Ethics Committee and written informed consent obtained from all the participating patients. We enrolled 60 patients aged between 18 and 60 years, all fit with the criteria of the American Society of Anaesthesiologists (ASA) Grade 3 undergoing elective OP-CABG. Patients who had contraindications to TxA, anticoagulation therapy, bleeding disorder, preoperative anaemia, and known hepatic/renal disease were excluded from the study.

We randomly recruited patients as per the inclusion/exclusion criteria in the intervention (I) group who received TxA during surgery till the desired sample size was obtained. Then we enrolled patients in the control (C) group (who didn’t receive TxA) by using an individual matching strategy. The matching criteria were based on age (+/- five years), gender, and operating procedure (OP-CABG). We continued to recruit patients till we achieved 30 matched pairs (I and C).

A standard protocol was followed for all the patients. Each patient admitted a day prior to surgery and a detailed history and examination were conducted. Patients on antiplatelets like clopidogrel and aspirin were advised to stop clopidogrel five days before surgery and to continue aspirin till the day of surgery. Routine blood investigations and coagulation profile were performed which were within normal limits for all the patients. The operative risk for the cardiac procedure was explained to patients according to the European System for Cardiac Operative Risk Evaluation II (EuroSCORE II) [5]. All patients were pre-medicated with tablet pantoprazole 40mg; tablet diazepam 5mg and tablet metoprolol 25 mg on the day of the surgery. On arrival to the operating room, standard ASA monitors were placed. General anaesthesia was administered with combination of intravenous (iv) midazolam 0.05-0.1 mg/kg, iv fentanyl 3-5 mcg/kg, pancuronium 0.1 mg/kg, and iv propofol 0.5-1.0 mg/kg titrated as per response. Iv TxA 10 mg/kg was administered at the time of induction only in the I group. Anaesthesia was maintained on volume-controlled ventilation with isoflurane, intermittent boluses of iv fentanyl 2 mcg/kg, and iv pancuronium 0.015 mg/kg. Depth of anaesthesia was monitored using the bispectral index. Postoperatively, patients were transferred to the cardiac intensive care unit (CICU) intubated and ventilated. Postoperative bleeding was measured in form of total chest drain collection every 24 hours till the chest drain was removed.

Statistical analysis

The sample size was calculated with the help of the study by Wang et al. [2] for a Type I error of 0.05 and a Type II error of 0.20 with a power equal to 80% and CI of 95%. The minimum required sample size was found to be 27 in each group. Thus, the total sample size was 60 patients with 30 patients in each group. The statistical analysis was performed using Statistical Package for the Social Sciences 22.0 version (SPSS, IBM Corp., Armonk, NY). Continuous variables were expressed as mean ± standard deviation and categorical variables as frequency and percentages. Shapiro-Wilk test was used to checking the normality. Student t-test or Mann Whitney U test was used for finding the significant mean difference between the ages, weight, EuroSCORE ІІ, total drain collection at 24 hours and 48 hours and is expressed as mean ± standard deviation.

## Results

Baseline demographic and preoperative parameters were comparable between both groups as depicted in Table 1. On the basis of sex distribution, the total number of males and female were 25 (83.3%) and 5 (16.7%) respectively in both groups as per matching criteria.

**Table 1:** Comparison of preoperative parameters between two groups n: number of study subjects; SD: standard deviation; HTN: hypertension; MI: myocardial infarction; EF: ejection fraction

Preoperative variable	Intervention group	Control group	p-Value
	(n total=30)	(n total=30)	
	Age (years, mean±2SD)	57.50±7.89		57.90±7.65	0.84
Weight (kg, mean±2SD)	71.9±3.73	73.46±3.23	0.35
EuroSCORE II	1.56±0.78	1.54±0.63	0.92
ASA grading (III)	30 (100)	30 (100)	
Diabetes, n (%)	21 (70)	16 (53.3)	0.28
HTN, n (%)	23 (76.7)	20 (66.7)	0.57
Smoker, n (%)	8 (26.7)	10 (33.3)	0.78
Renal impairment, n (%)	5 (16.7)	2 (6.7)	0.42
Stroke, n (%)	1 (3.3)	0	0.33
Angina, n (%)	8 (26.7)	5 (16.7)	0.36
Recent MI, n (%)	12 (40)	6 (20.0)	0.09
Lung disease, n (%)	0	0	
Pulmonary HTN, n (%)	0	0	
Arrhythmias, n (%)	0	0	
Extra cardiac arteriopathy, n (%)	0	0	
EF (%)	50.07±7.84	50.23±6.92	0.93

**Figure 1:**
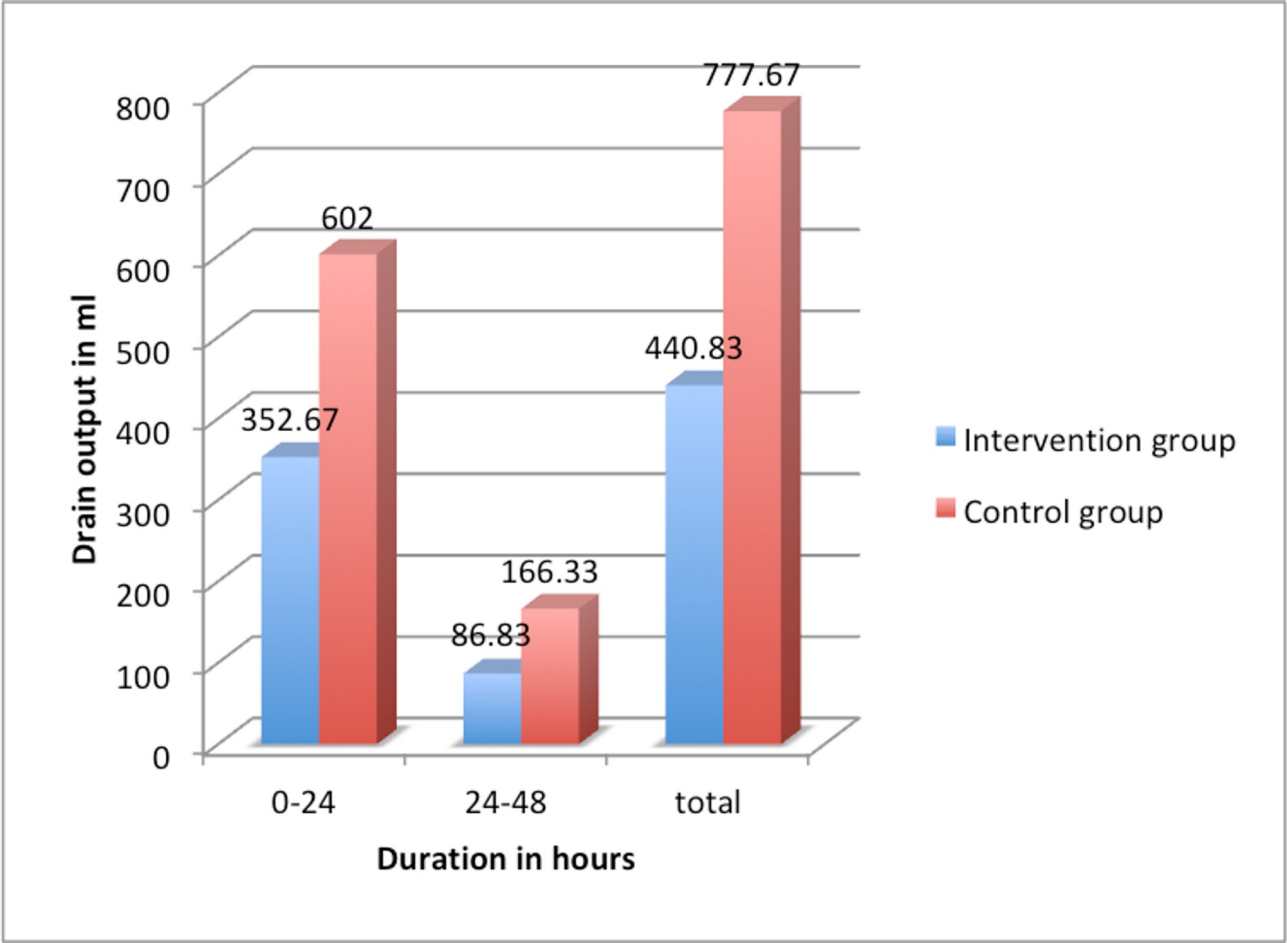
Comparison of postoperative blood loss among study groups

In our study, there was a significant reduction of postoperative chest drainage output in the intervention group in comparison to control group, showing the effectiveness of TxA in reducing postoperative blood loss (Table 2, Figure 1).

 

**Table 2:** Comparison of postoperative blood loss (chest drain output) between both groups t- t-test value; df- degree of freedom

Variables	Intervention group (ml) (n=30)	Control group (ml) (n=30)	Mean difference	95% CI	Statistical test (t, df, p-value)
Drainage 0-24 hours	352.67±97.84	602±188.58	249.33	(171.69,326.97)	(6.21, 58, <0.001)
Drainage 24-48 hours	86.83±76.66	166.33±112.57	79.5	(29.72,129.27)	(3.31, 58, 0.002)
Total drainage	440.83±155.96	777.67±277.88	336.84	(220.38,453.29)	(5.71, 58, <0.001)

Only one (3.30 %) patient required transfusion in the I group while five (16.70 %) in the C group which was statistically insignificant. Other postoperative variables were also comparable in both groups (Table 3). Postoperative ventilatory support, CICU and hospital stay was also recorded and showed comparable data among both groups depicting no significant effect of administering tranexamic acid in reducing the duration of ventilatory support, CICU and hospital stay (Table 3).

**Table 3:** Comparison of postoperative variables among study groups IABP: intra aortic balloon pump; MODS: multiple organ dysfunction syndrome; CICU: cardiac intensive care unit; IPPV: intermittent positive pressure ventilation

Postoperative variables	Intervention group (n=30)	Control group (n=30)	p-Value
Arrhythmias	0	0	
IABP	0	0	
Pacing n (%)	1 (3.3%)	0	
Re-exploration n (%)	0	0	
Blood transfusion n (%)	1 (3.3%)	5 (16.7%)	0.19
Perioperative MI n (%)	0	0	
CVA n (%)	0	0	
Infection n (%)	0	0	
Renal dysfunction n (%)	0	0	
MODS n (%)	0	0	
Cardiac arrest n (%)	0	0	
Death n (%)	0	0	
Cognition defect n (%)	0	0	
Inotropic support (hours)	24.13±6.74	24.47±5.69	0.84
CICU stay (hours)	49.73±16.07	47.20±10.94	0.47
IPPV duration (hours)	12.87±6.88	11.47±2.57	0.30
Hospital stay (days)	5.43±1.10	5.30±0.75	0.59

## Discussion

With the rising advent of interest in OP-CABG, various studies have been done to see the advantages of OP-CABG over on-pump CABG in terms of reducing bleeding, coagulopathy, and technically more efficacious [6-8]. The principal aim of avoiding the use of CPB is to eliminate its possible complications in the form of activation of coagulation, fibrinolysis, and perioperative inflammatory and haemorrhagic events. Although avoidance of CPB has been shown to significantly reduce perioperative bleeding and its related complications [9]. However, the risk is not eliminated in OP-CABG due to surgical trauma and the use of heparin-protamine [2,7]. These stimuli trigger a fibrinolytic cascade and increase the bleeding tendency.

Antifibrinolytic agents like aprotinin, epsilon-aminocaproic acid (EACA), and TxA have been studied in various randomized trials and meta-analysis for their efficacy in reducing perioperative blood loss in cardiac surgeries [10-14]. Aprotinin being a natural serine protease inhibitor; it has been proven to be associated with increased risks of adverse cardiovascular, cerebrovascular, and renal outcomes [12,13]. EACA inhibits fibrinolysis mainly by the inhibition of plasminogen inhibitors and to a lesser degree through antiplasmin activity but is 10 times less potent than TxA [11,12]. TxA, a lysin analogue, similar action like EACA being safer than other antifibrinolytics; it has been widely studied for its effects on reducing blood loss in on-pump CABG [10,12,15] but limited evidence in OP-CABG [2,3,9-11]. Thus, in our study, we decided to evaluate the effectiveness and safety of TxA in reducing postoperative blood loss in patients undergoing OP-CABG.

Baseline demographics, preoperative variables, and cardiac operative risk (euroSCORE II) were comparable among both groups as depicted in Table 1. Our results were consistent with the findings of Wang et al., Casati et al., and Vanek et al. [2,9,10].

The mean postoperative bleeding measured as chest drain output in the I group at 24 hours and 48 hours were 352.67 ml and 86.83 ml respectively. On the other hand, in the C group, it was 602.00 ml and 166.3 ml. The data showed a statistically significant difference in the postoperative chest drainage output between the groups (unpaired t-test, p < 0.05) and thus exhibiting a significant reduction in postoperative blood loss in the I group (Table 2, Figure 1). Casati et al. [9] studied the efficacy of TxA in OP-CABG and showed a significant reduction in postoperative bleeding in the first hour hours (150ml) in comparison to placebo (375ml) with p-value <0.0001 when the hemostatic effect of TxA is still present. Total bleeding also showed a significant reduction in the TxA group (p<0.0001). Similarly, Wang et al. [2] also showed a significant decrease in postoperative blood loss in the TxA group with 269.7 ml drain and 653.9 ml at the end of six hours and 24 hours respectively in comparison to the control group (p<0.001). In the study by Mehr-Aein et al. [3], the mean total drainage in the TxA group was 320 ml while in the control group 480ml. The statistical analysis showed a highly significant reduction in blood loss in the TxA group with a p-value < 0.001.

We have not found any statistically significant differences in transfusion requirements in both groups. In the I group only one patient (3.3%) was transfused while in the C group five patients (16.7%) received blood transfusion (Table 3). Our results were consistent with Vanek et al. [10]. However, Casati et al. [9] and Mehr-Aein et al. [3] found a lesser need for allogenic blood transfusion in the I group. In the study of Mehr-Aein and coworkers [3], 15% of patients were transfused in the TxA group while 36 % in the control groups which was highly significant with p < 0.05. The lack of statistical significance in our study was probably due to the small number of transfusion requirements in the study sample.

There was no postoperative morbidity and mortality in form of pulmonary dysfunction, neurological deficit, cardiac complications, and re-exploration (Table 3). The potential of antifibrinolytics in causing hypercoagulability was also taken into consideration. Thromboembolic complications can cause graft occlusion leading to MI. No evidence of hypercoagulable complications was detected in our study groups like deep vein thrombosis, pulmonary embolism, cerebrovascular accidents (Table 3). Our findings are consistent with Mehr-Aein et al. [3] and systemic review by Thiagarajamurthy et al. [11]. In our study, low dose TxA without the use of infusion also depicts the reduced risk of thromboembolic complications and seizure potentials. Lambert et al. [16] underwent a trial to find out complications of TxA at a low dose and high dose in CABG. There was a clear evident benefit of low dose tranexamic acid in reducing blood loss without any thromboembolic complications.

There are several limitations for this prospective study: (1) Relatively small cohort. (2) Patients were followed up only till the day of discharge from the hospital. (3) We could not randomise patients so followed matched cohort methodology to avoid bias. (4) Thromboembolic complications in form of deep vein thrombosis, pulmonary embolism were detected only on the basis of clinical events. Although the aforementioned limitations this is a prospective cohort study.

## Conclusions

There was a clear significant reduction in postoperative blood loss in TxA showing its utility in subsiding the fibrinolytic cascade efficiently. Thus this study indicates the use of TxA in OP-CABG effectively and safely to reduce postoperative blood loss.
